# The Influence of Anti-PAR 1 and Anti-ACE 2 Antibody Levels on the Course of Specific Glomerulonephritis Types

**DOI:** 10.3390/jcm14093178

**Published:** 2025-05-04

**Authors:** Maciej Szymczak, Harald Heidecke, Marcelina Żabińska, Łucja Janek, Jakub Wronowicz, Krzysztof Kujawa, Kai Schulze-Forster, Karolina Marek-Bukowiec, Tomasz Gołębiowski, Mirosław Banasik

**Affiliations:** 1 Clinical Department of Nephrology, Transplantation Medicine and Internal Diseases Wroclaw Medical University, 50-556 Wroclaw, Poland; karolina.marek-bukowiec@umw.edu.pl (K.M.-B.); tomasz.golebiowski@umw.edu.pl (T.G.); miroslaw.banasik@umw.edu.pl (M.B.); 2 CellTrend Gmbh, Im Biotechnologiepark 3 TGZ II, 14943 Luckenwalde, Germany; heidecke@celltrend.de (H.H.); schufo@celltrend.de (K.S.-F.); 3 Department of Preclinical Sciences, Pharmacology and Medical Diagnostics, Faculty of Medicine, Wrocław University of Science and Technology, 58-376 Wroclaw, Poland; marcelina.zabinska@pwr.edu.pl; 4 Statistical Analysis Center, Wroclaw Medical University, 50-368 Wroclaw, Poland; lucja.janek@umw.edu.pl (Ł.J.); jakub.wronowicz@umw.edu.pl (J.W.); krzysztof.kujawa@umw.edu.pl (K.K.)

**Keywords:** glomerulonephritis, antibodies

## Abstract

**Background:** Anti-PAR 1 (protease-activated receptor 1) and anti-ACE 2 (angiotensin 2-converting enzyme 2) antibodies are a kind of non-HLA (human leukocyte antigens) antibodies postulated to be of significance in autoimmunological diseases and organ transplantation. **Methods:** We assessed anti-PAR 1 and anti-ACE 2 antibody levels in patients with membranous nephropathy *n*= 18, focal and segmental glomerulosclerosis (FSGS) *n* = 25, lupus nephritis (LN) *n* = 17, IgA nephropathy *n* = 14, mesangial proliferative (non-IgA) glomerulonephritis *n* = 6, c-ANCA (cytoplasmic anti-neutrophil cytoplasmic antibodies) vasculitis *n* = 40, p (perinuclear)-ANCA vasculitis *n* = 16, and compared them with a healthy control group *n* = 22. Next, we observed the clinical course of the patients (creatinine, total protein, and albumin) up to 2 years and correlated the results with the level of antibodies. **Results:** The anti-PAR 1 antibody level was lower in membranous nephropathy and FSGS compared to the control group. Anti-PAR 1 antibody levels were higher in secondary compared to primary glomerulonephritis. Both anti-PAR 1 and anti-ACE 2 antibody levels correlated positively (in focal and segmental glomerulosclerosis) or negatively (in lupus nephritis) with total protein and albumin at different time points of observation. Anti-PAR 1 and anti-ACE 2 antibody levels correlated also with creatinine level at one time point of observation in IgA nephropathy. Anti-PAR 1 and anti-ACE 2 antibodies correlated with each other in membranous nephropathy, FSGS, and p- and c-ANCA vasculitis (*p* < 0.05). **Conclusions:** The anti-PAR 1 antibody level was lower in membranous nephropathy and focal and segmental glomerulosclerosis compared to the control group. Anti-PAR 1 antibody levels tend to be higher in secondary compared to primary glomerulonephritis.

## 1. Introduction

Glomerular diseases are autoimmune disorders connected with immune system activation and the appearance of autoantibodies. Some autoantibodies are markers for glomerulonephritis prognosis, although the significance of specific isotypes differs between the types of glomerulonephritis [1]. Many autoantibodies are classified as non-human leukocyte antigen (HLA) antibodies [2], such as those raised against angiotensin 2 type 1 receptor (AT1R), angiotensin 2 type 2 receptor (AT2R), and endothelin A receptor (ETAR), all of which have some significance in organ transplantation rejection [3] and glomerulonephritis development [4].

Some markers are connected with specific molecular pathways, and these pathways can influence each other. Protease-activated receptor type 1 (PAR 1) binds thrombin [5], and angiotensin-converting enzyme type 2 (ACE 2) [6] is connected with the renin–angiotensin system, so these proteins are linked to different pathways. There are some data on the role of these proteins in kidney diseases, but knowledge of antibodies against PAR 1 and ACE 2 in kidney diseases is still lacking.

PAR 1 is expressed on podocytes, the endothelium, and epithelial renal cells [7] and it is activated by thrombin and factor Xa [8]. PAR 1 activation induces vasoconstriction [9], inhibits renin [10], plays a role in platelet activation and aggregation [11], and facilitates endothelial migration [12]. As such, PAR 1 inhibition protects the endothelium and ameliorates vascular injury [13].

PAR 1 deficiency protects against the development of streptozotocin-induced diabetes in experimental mouse models [14], while some data indicate that PAR 1 blockers alleviate nephrotoxicity in fibrosis, tubular inflammation, and mitochondrial dysfunction [15]. On the other hand, PAR 1 activation in human proximal tubule epithelial cells decreases the expression of transforming growth factor-beta (TGF-β) and diminishes tumour necrosis factor-alpha (TNF-α)-induced interleukin 6 (IL-6) and IL-8 production [16].

PAR 1 participates in the development of renal inflammation during the course of crescentic glomerulonephritis [17], while PAR 1 activation in mice produces a model of focal and segmental glomerulosclerosis (FSGS) [18].

The activation or inhibition of PAR 1 caused a deterioration in kidney function in a rat model, while normal PAR 1 resulted in the optimal preservation of kidney function [19]. However, not all findings from rat models apply to humans [20]. PAR 1-mediated calcium oscillations in mesangial cells were shown to influence cellular proliferation [21].

Autoantibodies against PAR 1 were found in patients with scleroderma renal crisis and induced IL-6 production, which indicates an immunomodulatory response rather than a strictly agonistic or antagonistic effect [22]. High levels of anti-PAR 1 antibodies were also found in cases of acute rejection following hand transplantation [23] and severe coronavirus disease 2019 (COVID-19), with very high levels in those with thrombotic complications [24].

ACE 2 is a monocarboxypeptidase that degrades angiotensin 2 to angiotensin (1–7) [25], vasodilators with anti-fibrotic activity [26]. ACE 2 is expressed in kidney glomeruli (especially podocytes and mesangial cells) [27], proximal tubules, collecting ducts, and the vasa recta [28].

Circulating ACE 2 was identified as a cardiovascular risk factor in males with chronic kidney disease, with elevated levels found in cardiovascular diseases, diabetes, and hypertension. ACE 2 levels increased with kidney function deterioration in diabetic patients and correlated with urinary protein excretion [29].

The method for assessing anti-ACE 2 antibodies was first described in 2013, and since the antibodies are antagonistic, they neutralize ACE 2 activity [30]. Indeed, anti-ACE 2 antibodies blocked severe acute respiratory syndrome coronavirus 2 (SARS-COV2) from binding [31]. However, their presence in anti-melanoma differentiation-associated gene 5 antibody-positive dermatomyositis (MDA5-DM) worsened prognosis (this case involved IgM antibodies; we evaluated IgG antibodies) [32].

The aim of our study was to evaluate anti-PAR 1 and anti-ACE 2 antibody levels in different glomerular diseases, to check the existence of associations between these antibodies and clinical data, and to find out if these antibodies could be a good candidate for future studies evaluating their prognostic potential in the course of specific diseases.

The relationships between anti-PAR 1 and anti-ACE 2 antibody levels in glomerular disorders were evaluated in the current investigation. In order to examine the relationship between initial antibody levels and the clinical progression of particular diseases, we also recorded the clinical outcomes of patients throughout a two-year period following the antibody evaluation.

## 2. Materials and Methods

**Figure 1:**
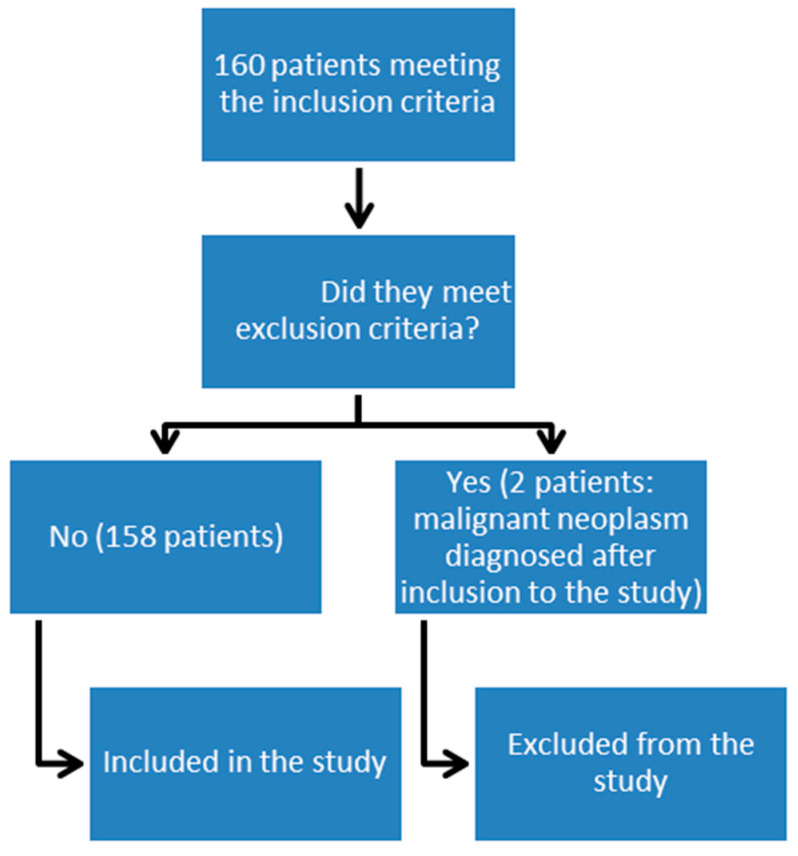
A flowchart illustrating the procedure for recruitment into the study.

The study recruited patients visiting our clinic between 2013 and 2020 with the first signs and symptoms of disease or relapse, and they were enrolled before commencing any intensive treatment (e.g., cyclophosphamide and steroid pulses). All the patients in the study received no drugs at the moment of inclusion and within the preceding 6 months. Due to the study design, there was no randomization. Only patients with a histopathologically confirmed diagnosis of membranous nephropathy, FSGS (focal and segmental glomerulosclerosis), LN (lupus nephritis), IgA nephropathy, non-IgA mesangial proliferative glomerulopathy, cytoplasmic (c-ANCA) or perinuclear (p)-ANCA-positive anti-neutrophil cytoplasmic antibody vasculitis were included. Moreover, proteinuria in the last accessible urine evaluation was required for the patient to be included. Patients with a history of current or previous malignancy, dialysis, or kidney transplantation, and those with active infections were excluded from the study. The control group comprised 22 healthy individuals of Caucasian origin who met the criteria of having no proteinuria, creatinine levels at or below 1.3 mg/dL, CRP levels under 5 mg/dL, and no history of kidney, autoimmune, or neoplastic disorders in anamnesis. Figure 1 displays a flowchart outlining the process of participant inclusion in the study.

Every group of patients was marked with a number: 1—membranous nephropathy, 2—focal and segmental glomerulosclerosis, 3—lupus nephritis, 4—IgA nephropathy, 5—non-IgA mesangial proliferative glomerulonephritis, 6—control group, 7—c-ANCA-positive vasculitis, and 8—p-ANCA-positive vasculitis.

Before the materials were collected, each participant in the study—all of whom were from Poland—signed an informed consent form. The study received approval from Wroclaw Medical University’s Bioethics Committee: No. KB-221/2023 and KB-546/2012.

Serum was obtained from 158 healthy controls and patients with certain glomerular disorders: membranous nephropathy (*n* = 18), FSGS (*n* = 25), lupus nephritis (LN) (*n* = 17), IgA nephropathy (*n* = 14), non-IgA mesangial proliferative glomerulonephritis (*n* = 6), c-ANCA-positive vasculitis (*n* = 40), p-ANCA-positive vasculitis (*n* = 16), and membranous nephropathy (*n* = 18) were among the patient diagnoses.

Using a second sample tube and no further venepuncture, 2.7 mL of blood was drawn from the patients in addition to blood drawn for routine laboratory analysis. Fasting the morning before the vein puncture was not required of the patients.

The lab was situated in close proximity to the clinical ward, allowing for the rapid transportation of materials. The centrifugation of samples started precisely 10 min after the collection to avoid any bias from varying material storage durations prior to freezing. Blood samples were not taken when the temperature exceeded 28 °C. The blood was centrifuged at 1500 g for a duration of 10 min, after which serum was extracted and stored at −80 °C.

The serum concentrations of anti-PAR 1 and anti-ACE 2 antibodies were evaluated using commercially available enzyme-linked immunosorbent assay (ELISA) kits (CellTrend, Luckenwalde, Germany) following the manufacturer’s instructions: anti-PAR 1 antibody kit (catalogue number 12200) and anti-ACE 2 antibody kit (catalogue number 16000). Microtiter plates were pre-coated with PAR 1 and ACE 2, to which 1:100 dilution samples were added and incubated for two hours at 2–8 °C. After the washing steps, anti-PAR 1 and anti-ACE 2 antibodies were detected with horseradish peroxidase (POD)-labelled anti-human IgG (1:100), which was developed with a 3, 3′, 5, 5′-tetramethylbenzidine (TMB) substrate solution. Measurements were performed at 450 nm with a correction wavelength of 630 nm, with the optical density then converted to a concentration using a standard curve. A value ≥ 2.5 U/mL was considered a positive result, and <2.5 U/mL was considered negative for the anti-PAR 1 antibody. Meanwhile, a concentration of ≥6.0 U/mL indicated a positive result for anti-ACE 2, and <6.0 U/mL was negative.

ELISAs were validated in accordance with the Food and Drug Administration (FDA) Bioanalytical Method Validation Guidance for Industry. There were no significant cross-reactions with other lupus antibodies.

Clinical data were collected for serum creatinine, eGFR (glomerular filtration rate—MDRD formula—Modification of Diet in Renal Disease), BUN (blood urea nitrogen), proteinuria, albumin/creatinine urine ratio, total serum protein, and serum albumin at baseline and after one, three, six, twelve, and twenty-four months, with age and sex also recorded. We also noted basic levels of leukocytes, hemoglobin, hematocrit.

The Kruskal–Wallis test, Dunn’s test with Bonferroni correction, or analysis of variance (ANOVA) compared anti-PAR 1 and anti-ACE 2 antibody concentrations, serum creatinine, eGFR, BUN, serum albumin, serum total protein, proteinuria, albumin/creatinine ratio, age, and sex between the glomerulonephritis and control groups.

Correlation analysis between quantitative variables employed Pearson’s or Spearman’s correlation coefficient, following a Shapiro–Wilk test to determine data distribution. The significance of correlations was determined using a t-test, with *p* < 0.05 signifying a positive result. The correlation analysis compared anti-PAR 1 and anti-ACE 2 antibody levels with clinical data. Additional comparisons included the levels of ANA (antinuclear antibodies) in LN patients, c-ANCA in granulomatosis with polyangiitis patients, and p-ANCA in p-ANCA-positive vasculitis patients with anti-PAR 1 and anti-ACE 2 antibody concentrations.

Spearman’s correlation evaluated the variability of clinical factors (serum creatinine, eGFR, BUN, serum albumin, and serum total protein levels) over the two-year observation period for all groups based on initial anti-PAR 1 and anti-ACE 2 antibody levels. The analysis included the evaluation of the statistical range, standard deviation, coefficient of variation, and trends in antibody levels over time. All analyses employed STATISTICA 13.

## 3. Results

### 3.1. Characteristics of Patients

The clinical parameters did not differ statistically between the specific groups, apart from some cases which are specified below.

Creatinine levels were higher in the p-ANCA-positive vasculitis patients than in the control group (*p* < 0.05). BUN was higher and eGFR lower in p-ANCA vasculitis groups compared to all other groups (*p* < 0.05). Patients from the control group had higher total protein and albumin levels than the other groups (*p* < 0.05). Patients from groups with specific diseases had proteinuria and patients from the control group had no proteinuria.

The hematocrit level in the c-ANCA vasculitis group was lower than that of the control group (*p* < 0.05).

Table 1 and Table 2 outline the patient clinical data.

### 3.2. Anti-PAR 1 Antibody Evaluation Results

Anti-PAR 1 antibody levels were lower in those with membranous nephropathy (*n* = 18) (*p* = 0.02) and focal and segmental glomerulosclerosis (*n* = 25) (*p* = 0.003) compared to the control group (*n* = 22).

**Figure 2:**
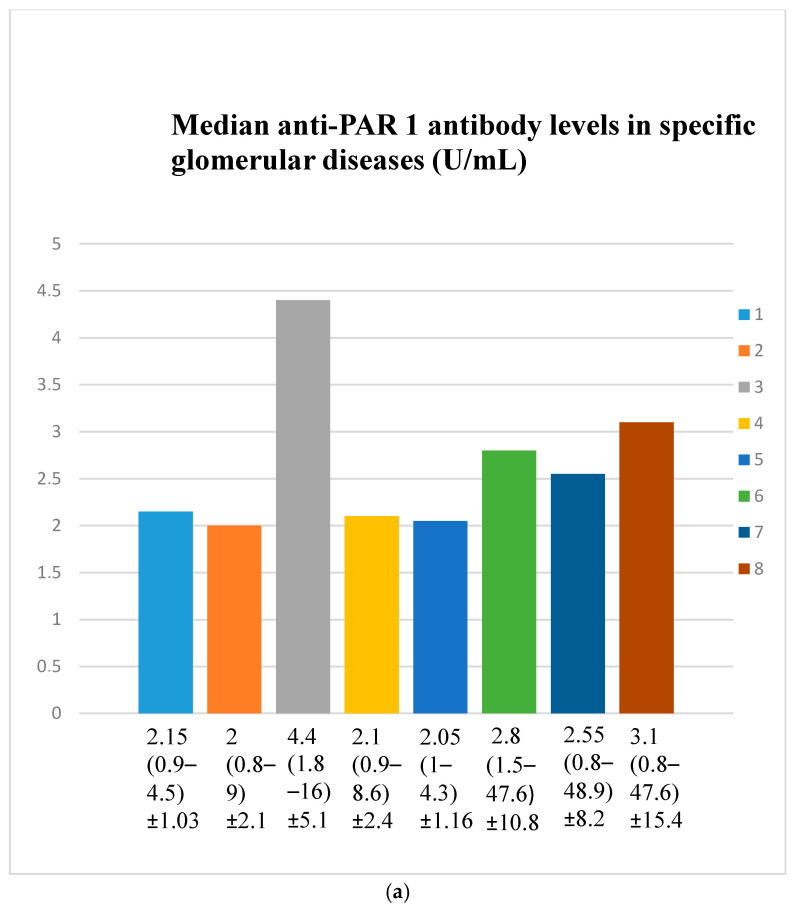

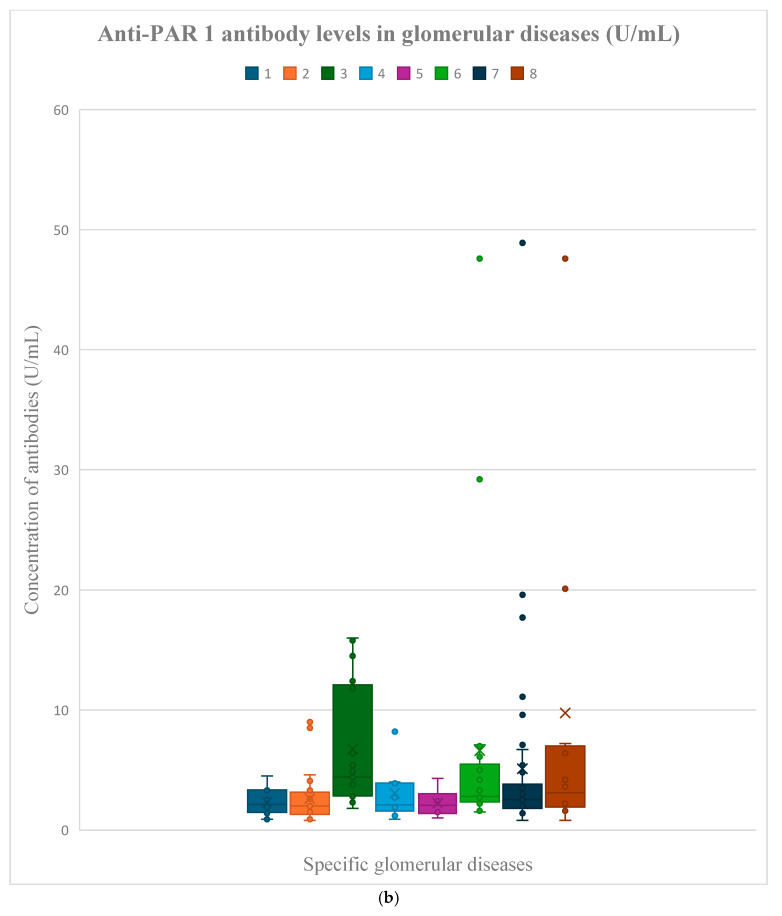
(**a**). Median anti-PAR 1 antibody levels in specific glomerular diseases (U/mL). (**b**). Anti-PAR 1 antibody levels in specific glomerular diseases (U/mL). The bars with colours labelled by numbers 1–8 represent values of anti-PAR 1 antibodies in patient groups 1–8, respectively (the same groups as in Figure 2a). The y-axis represents the concentration of antibodies (U/mL). Points represents specific patients. X-presents median value. Key for the (**a**,**b**): 1—membranous nephropathy (*n* = 18); 2—focal and segmental glomerulosclerosis (*n* = 25); 3—lupus nephritis (*n* = 17); 4—IgA nephropathy (*n* = 14); 5—mesangial proliferative (non-IgA) glomerulonephritis (*n* = 6); 6—control group (*n* = 22); 7—c-ANCA vasculitis (*n* = 40); and 8—p-ANCA vasculitis (*n* = 16). Median values are presented under the bars, ranges are values in brackets, and ± represents the standard deviation.

Anti-PAR 1 antibody levels were higher in lupus nephritis (*n* = 17) patients compared to those with membranous nephropathy (*n* = 18) (*p* = 0.0004), focal and segmental glomerulosclerosis (*n* = 25) (*p* = 0.00003), IgA nephropathy (*n* = 14) (*p* = 0.004), non-IgA mesangial proliferative glomerulonephritis (*n* = 6) (*p* = 0.007), and c-ANCA-positive vasculitis (*n* = 40) (*p* = 0.01) (Figure 2a,b). Anti-PAR 1 antibody values in particular patients of specific groups are presented in Figure S1.

A comparison of primary glomerulonephritis (membranous nephropathy, focal and segmental glomerulosclerosis, IgA nephropathy, mesangio-capillary non-IgA glomerulonephritis) and secondary glomerulonephritis (lupus nephritis, c-ANCA vasculitis, and p-ANCA vasculitis) indicates that the anti-PAR 1 antibody level was lower in primary glomerulonephritis: median 2 U/mL (range: 0.8–8.6) compared to secondary glomerulonephritis median 3 U/mL (range: 0.8–48.9) (*p* = 0.002).

### 3.3. Anti-ACE 2 Antibody Evaluation Results

**Figure 3:**
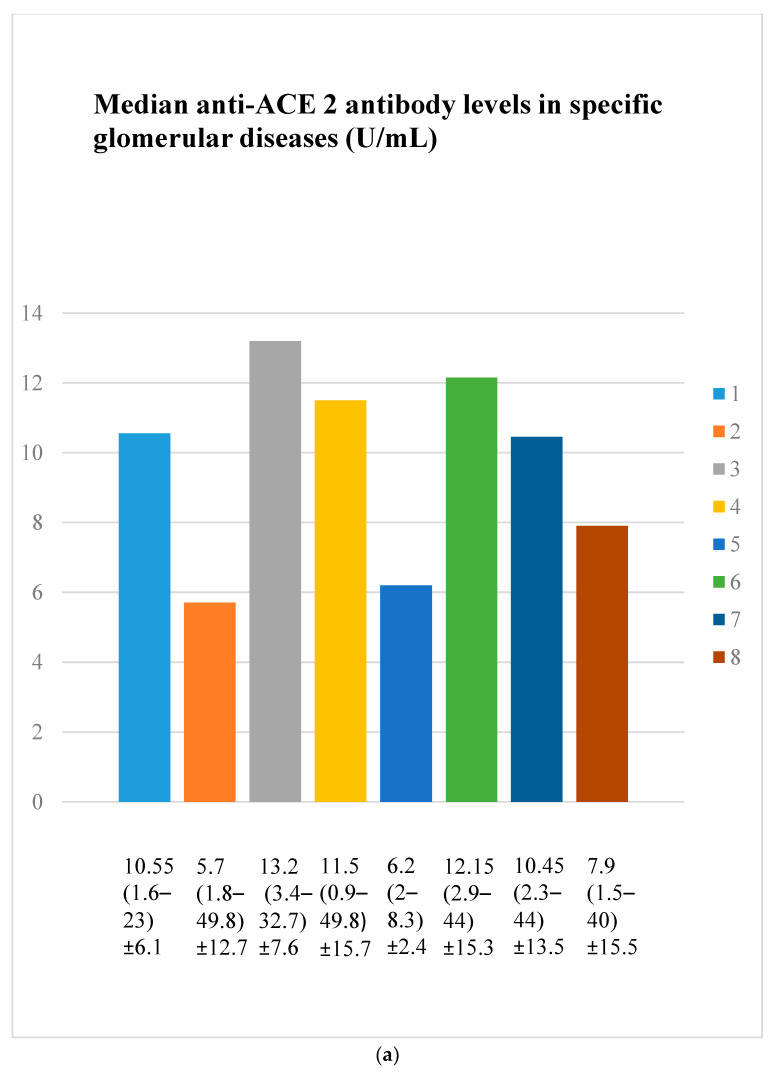

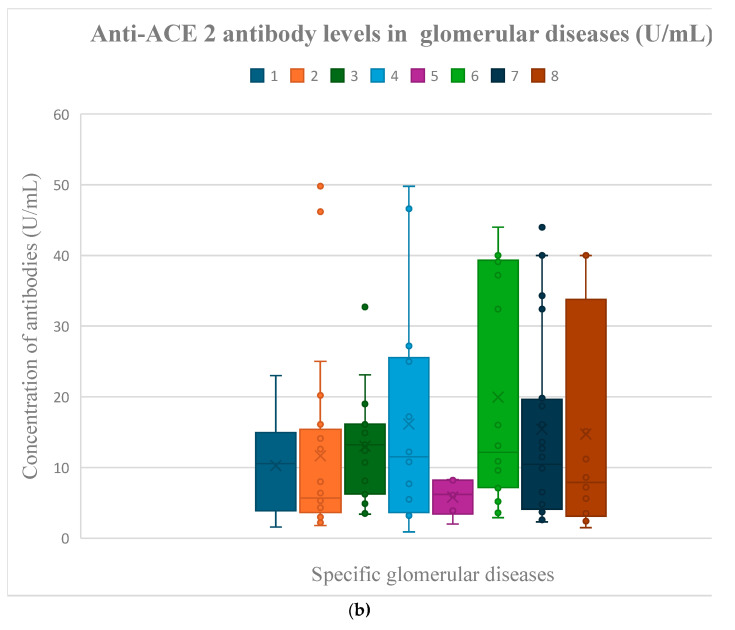
(**a**). Median anti-ACE 2 antibody levels in specific glomerular diseases (U/mL). (**b**). Anti-ACE 2 antibody levels in specific glomerular diseases (U/mL). The bars with colours labelled by numbers 1–8 represent values of anti-ACE 2 antibodies in patient groups 1–8, respectively (the same groups as in Figure 2a). The y-axis represents the concentration of antibodies (U/mL). Points represents specific patients. X-presents median value.

Anti-ACE 2 antibody levels did not differ statistically significantly between the groups (Figure 3a,b).

Anti-ACE 2 antibody values in particular patients of specific groups are presented in Figure S2.

### 3.4. Correlations Between Anti-PAR 1 Antibody Levels and Clinical Data of Patients

**Figure 4:**
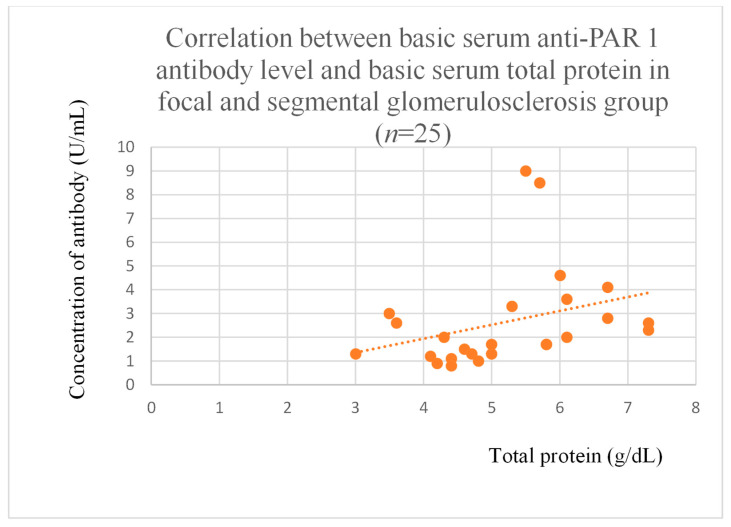
Correlation between basic serum anti-PAR 1 antibody level and basic serum total protein level in focal and segmental glomerulosclerosis group (r = 0.52; *p* = 0.009). Orange dots represent particular patients. Orange dotted line—trend line.

**Figure 5:**
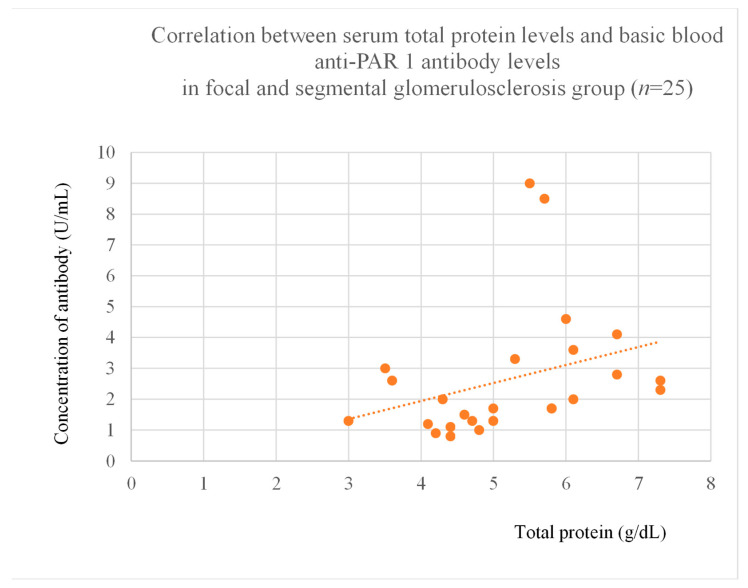
Correlation between basic serum anti-PAR 1 antibody level and serum albumin level after 1 month of observation in focal and segmental glomerulosclerosis group (r = 0.43; *p* = 0.03). Orange dots represent particular patients. Orange dotted line—trend line.

**Figure 6:**
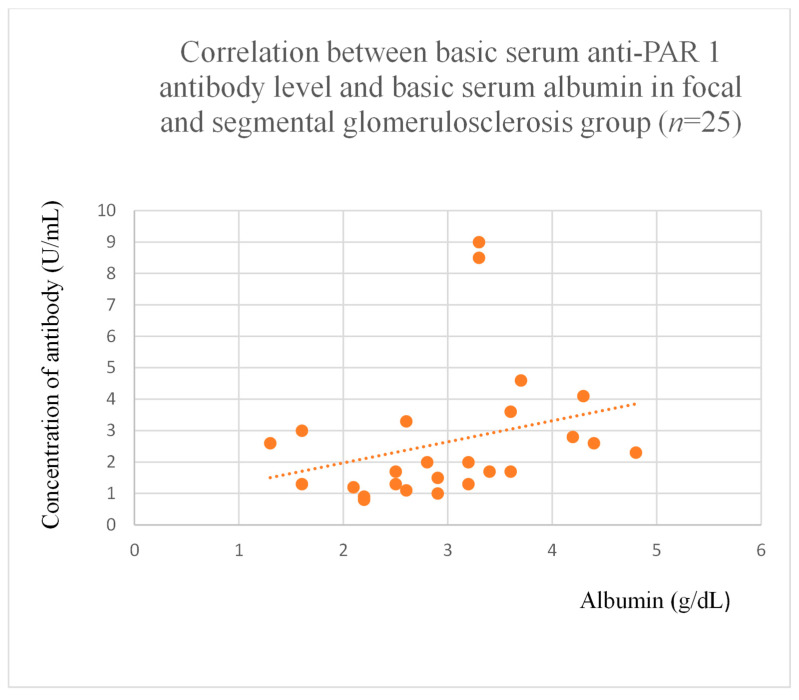
Correlation between basic serum anti-PAR 1 antibody level and basic serum albumin level in focal and segmental glomerulosclerosis group (r = 0.49; *p* = 0.01). Orange dots represent particular patients. Orange dotted line—trend line.

**Figure 7:**
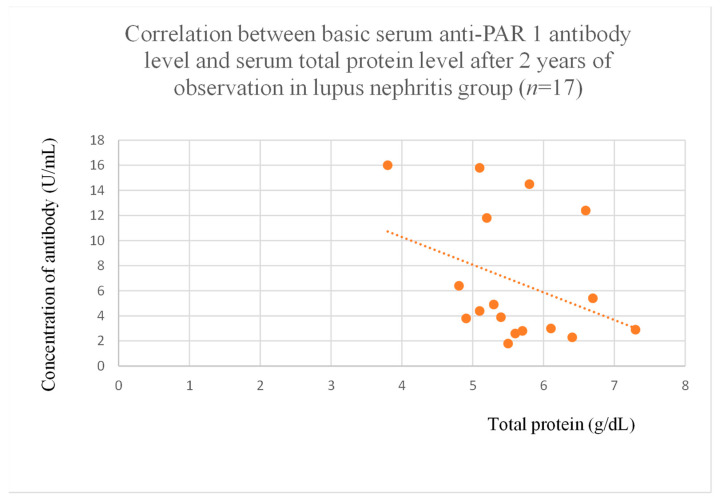
Correlation between basic serum anti-PAR 1 antibody level and serum total protein level after 2 years of observation in lupus nephritis group (r = −0.64; *p* = 0.005). Orange dots represent particular patients. Orange dotted line—trend line.

**Figure 8:**
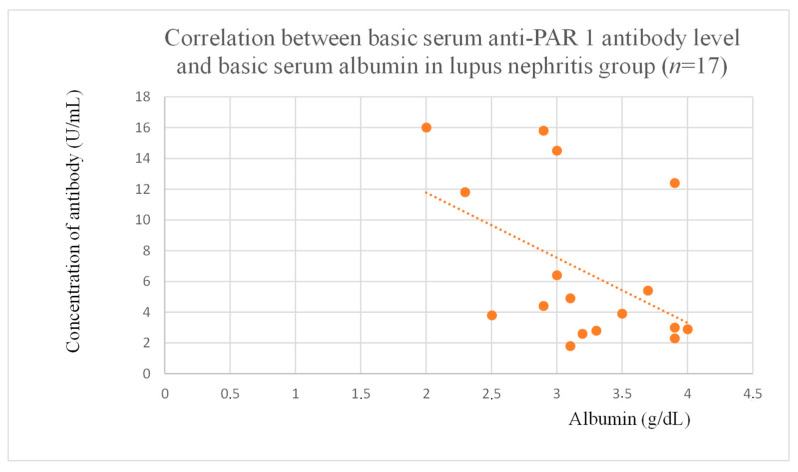
Correlation between basic serum anti-PAR 1 antibody level and serum albumin level in lupus nephritis group (r = −0.5; *p* = 0.04). Orange dots represent particular patients. Orange dotted line—trend line.

**Figure 9:**
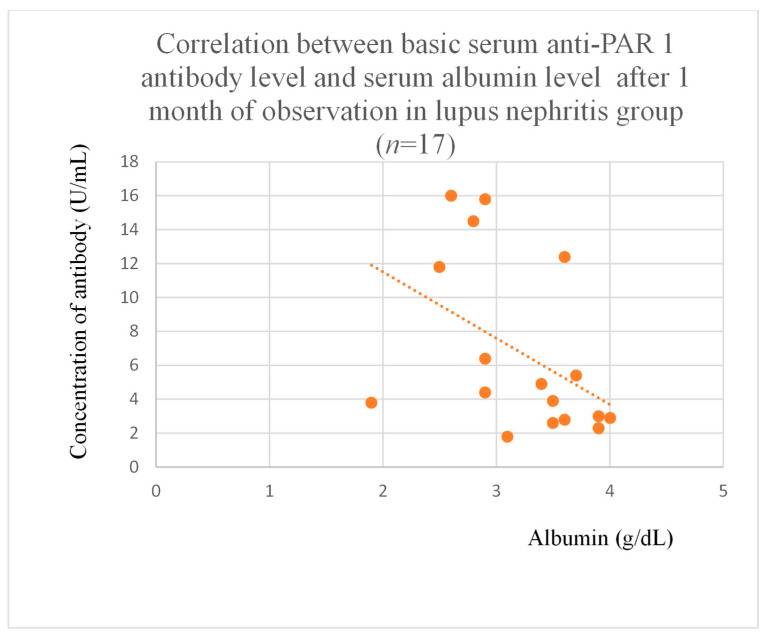
Correlation between basic serum anti-PAR 1 antibody level and serum albumin level after 1 month of observation in lupus nephritis group (r = −0.52; *p* = 0.03). Orange dots represent particular patients. Orange dotted line—trend line.

**Figure 10:**
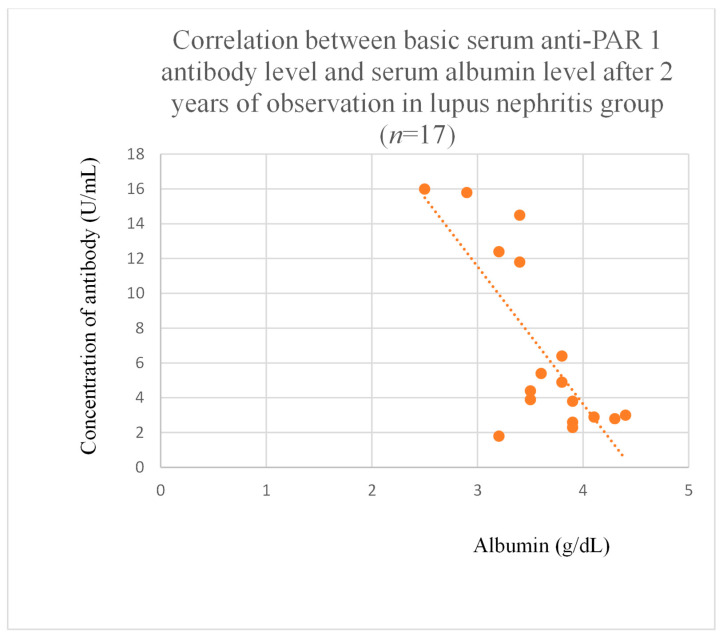
Correlation between basic serum anti-PAR 1 antibody level and serum albumin level after 2 years of observation in lupus nephritis group (r = −0.66; *p* = 0.004). Orange dots represent particular patients. Orange dotted line—trend line.

**Figure 11:**
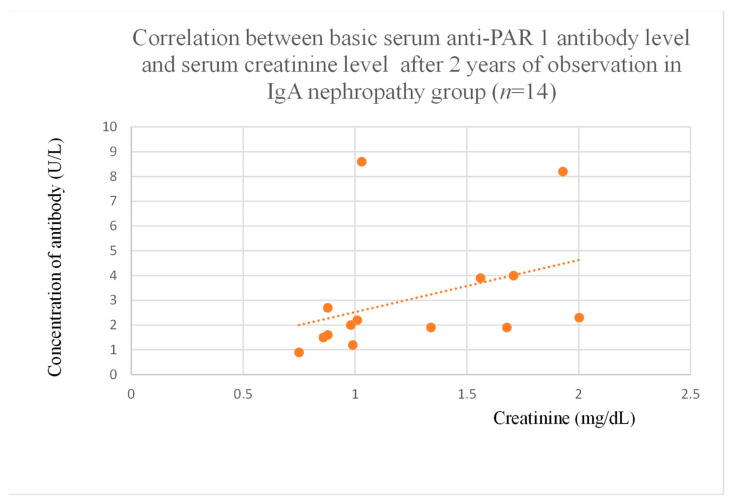
Correlation between basic serum anti-PAR 1 antibody level and serum creatinine level after 2 years of observation in IgA nephropathy group (r = 0.63; *p* = 0.02). Orange dots represent particular patients. Orange dotted line—trend line.

Anti-PAR 1 antibody levels correlated with total protein at baseline (Figure 4) and after one month of observation (Figure 5), and baseline albumin (Figure 6) in the focal and segmental glomerulosclerosis group. Moreover, anti-PAR 1 inversely correlated with total protein after two years of observation (Figure 7) and albumin at baseline (Figure 8), after one month (Figure 9) and two years of observation (Figure 10) in the lupus nephritis group. Baseline anti-PAR 1 antibody levels and creatinine were also correlated after two years of observation in the IgA nephropathy group (Figure 11). 

### 3.5. Correlations Between Anti-ACE 2 Antibody Levels and Clinical Data of Patients

**Figure 12:**
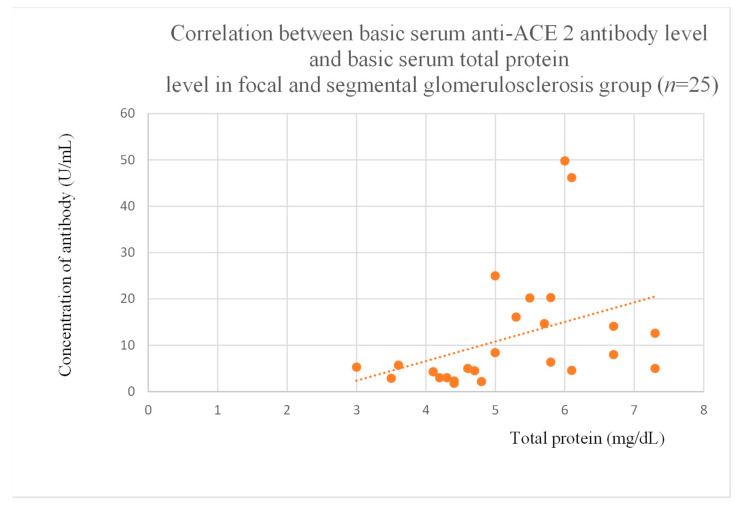
Correlation between basic serum anti-ACE 2 antibody level and basic serum total protein level in focal and segmental glomerulosclerosis group (r = 0.56; *p* = 0.003). Orange dots represent particular patients. Orange dotted line—trend line.

**Figure 13:**
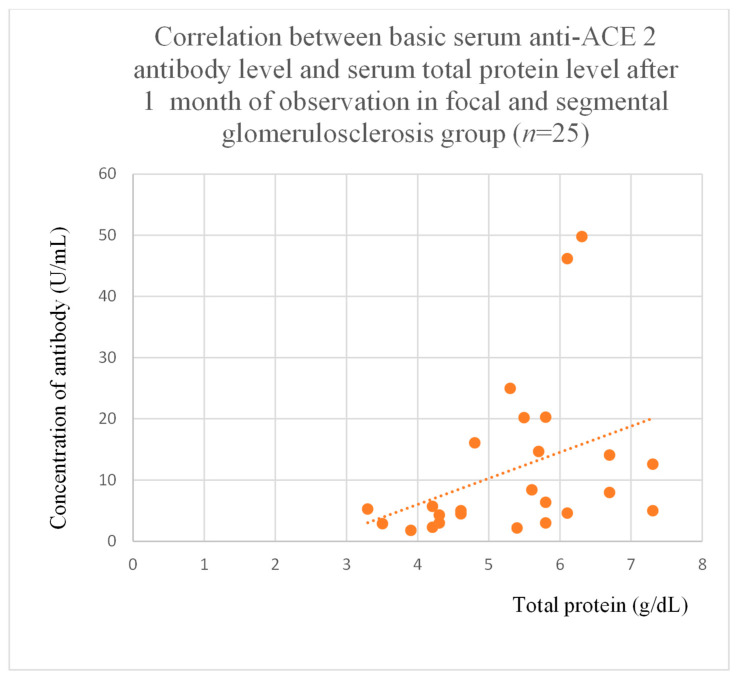
Correlation between basic serum anti-ACE 2 antibody level and serum total protein level after 1 month of observation in focal and segmental glomerulosclerosis group (r = 0.47; *p* = 0.03). Orange dots represent particular patients. Orange dotted line—trend line.

**Figure 14:**
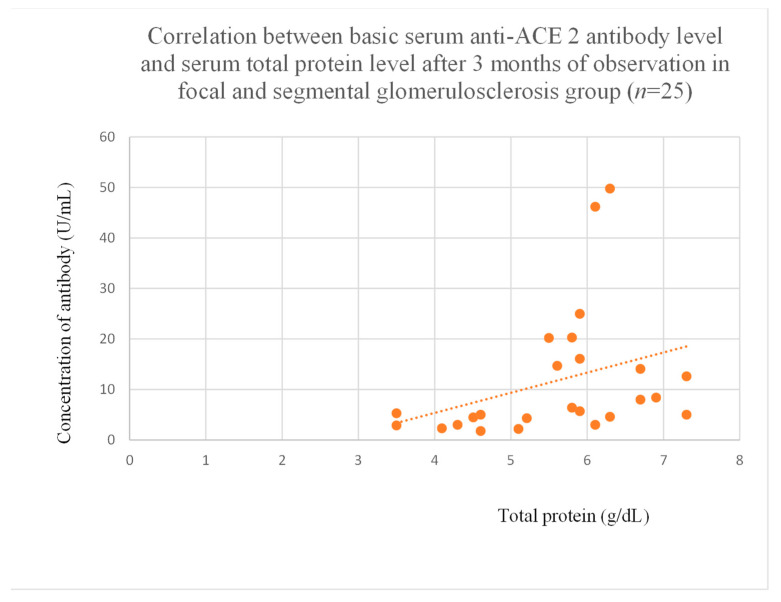
Correlation between basic serum anti-ACE 2 antibody level and serum total protein level after 3 months of observation in focal and segmental glomerulosclerosis group (r = 0.47; *p* = 0.03). Orange dots represent particular patients. Orange dotted line—trend line.

**Figure 15:**
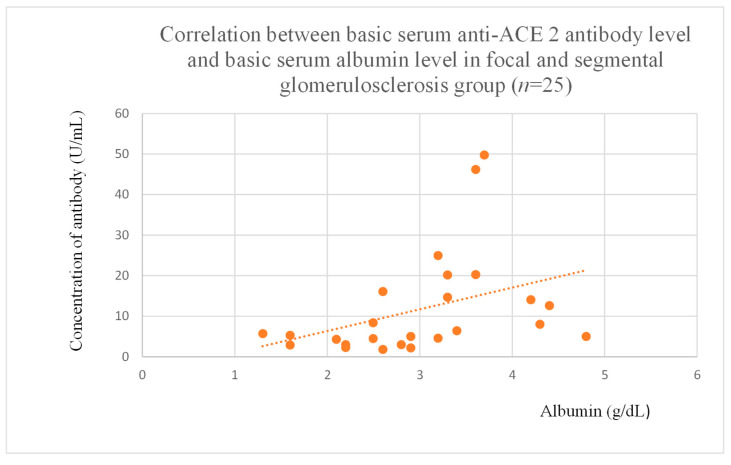
Correlation between basic serum anti-ACE 2 antibody level and basic serum albumin level in focal and segmental glomerulosclerosis group (r = 0.54; *p* = 0.004). Orange dots represent particular patients. Orange dotted line—trend line.

**Figure 16:**
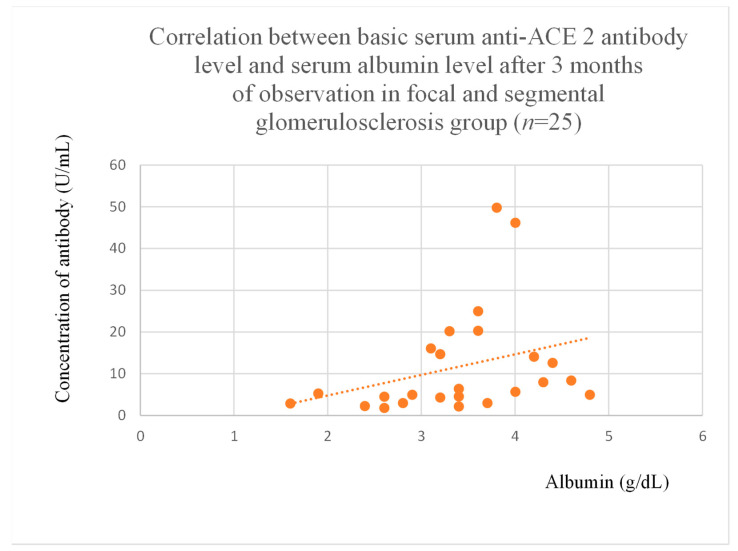
Correlation between basic serum anti-ACE 2 antibody level and serum albumin level after 3 months of observation in focal and segmental glomerulosclerosis group (r = 0.45; *p* = 0.04). Orange dots represent particular patients. Orange dotted line—trend line.

**Figure 17:**
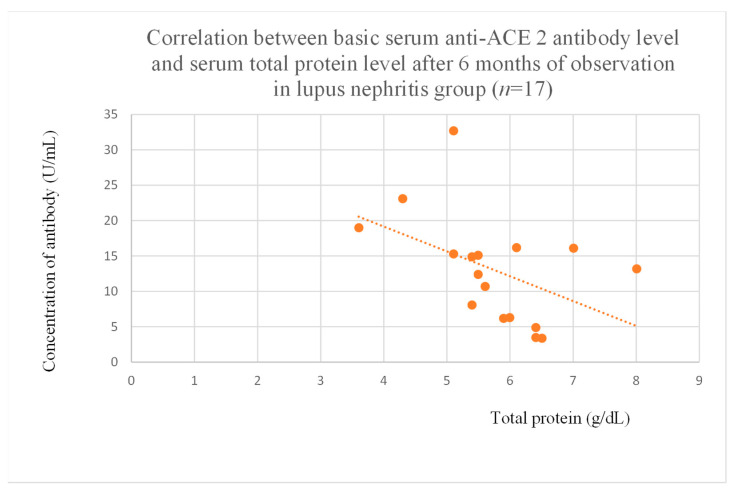
Correlation between basic serum anti-ACE 2 antibody level and serum total protein level after 6 months of observation in lupus nephritis group (r = −0.65; *p* = 0.01). Orange dots represent particular patients. Orange dotted line—trend line.

**Figure 18:**
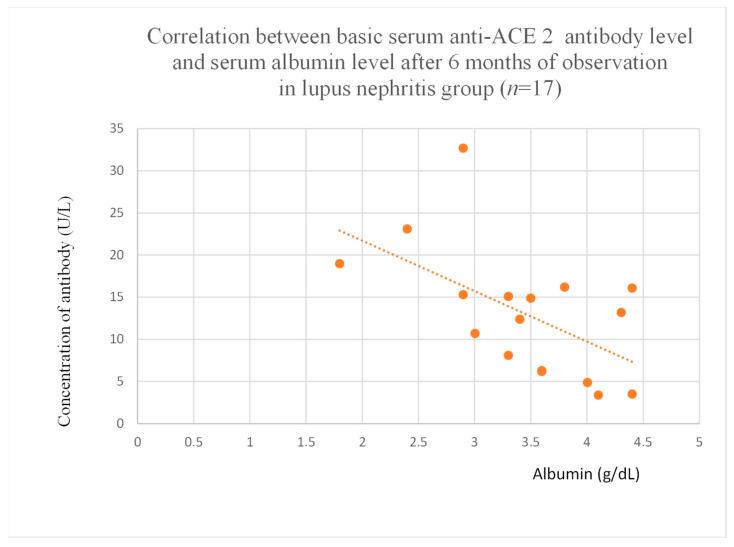
Correlation between basic serum anti-ACE 2 antibody level and serum albumin level after 6 months of observation in lupus nephritis group (r = −0.68; *p* = 0.009). Orange dots represent particular patients. Orange dotted line—trend line.

Anti-ACE 2 antibody levels correlated with total protein at baseline (Figure 12) after one month (Figure 13), and three months (Figure 14) of observation, and with albumin at baseline (Figure 15) and after three months of observation (Figure 16) in the focal and segmental glomerulosclerosis group. Moreover, anti-ACE 2 inversely correlated with total protein (Figure 17) and albumin (Figure 18) after six months of observation in the lupus nephritis group.

**Figure 19:**
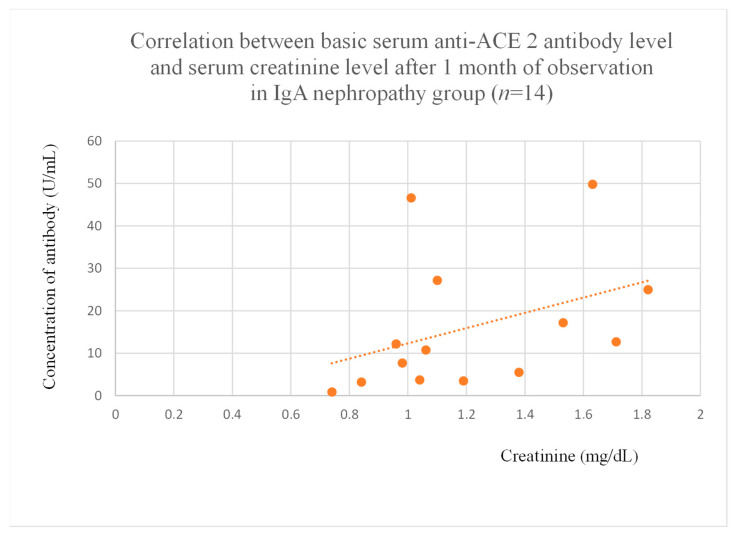
Correlation between basic serum anti-ACE 2 antibody level and serum creatinine level after 1 month of observation in IgA nephropathy group (r = 0.55; *p* = 0.04). Orange dots represent particular patients. Orange dotted line—trend line.

The authors also found a significant correlation between baseline anti-ACE 2 antibody levels and creatinine after one month of observation in the IgA nephropathy group (Figure 19).

### 3.6. Correlations Between Anti-PAR 1 and Anti-ACE 2 Antibody Levels

**Figure 20:**
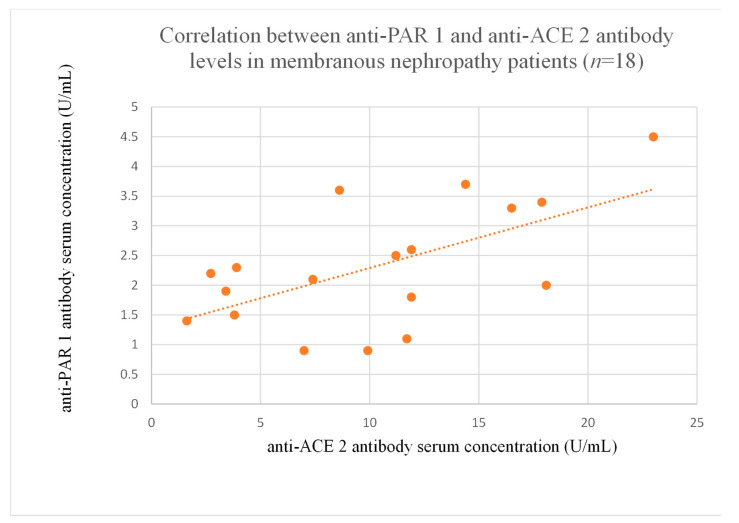
Correlation between anti-PAR 1 and anti-ACE 2 antibody levels in membranous nephropathy patients (r = 0.51, *p* = 0.03).

**Figure 21:**
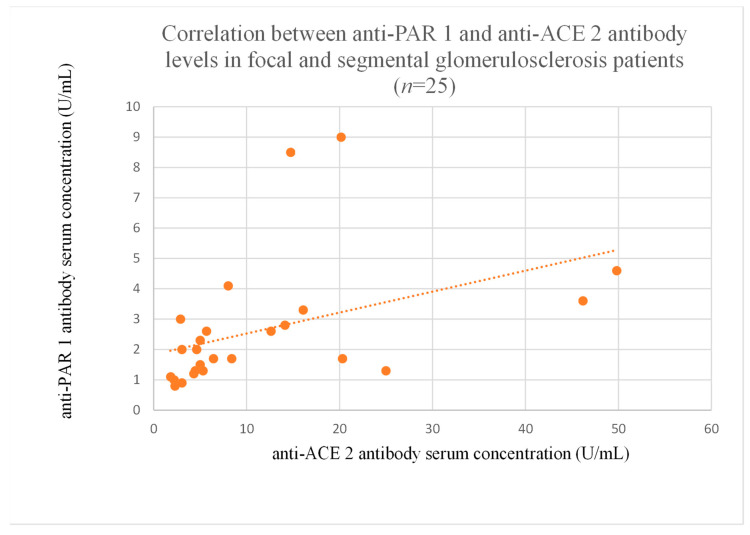
Correlation between anti-PAR 1 and anti-ACE 2 antibody levels in focal and segmental glomerulosclerosis patients (r = 0.65, *p* = 0.001).

**Figure 22:**
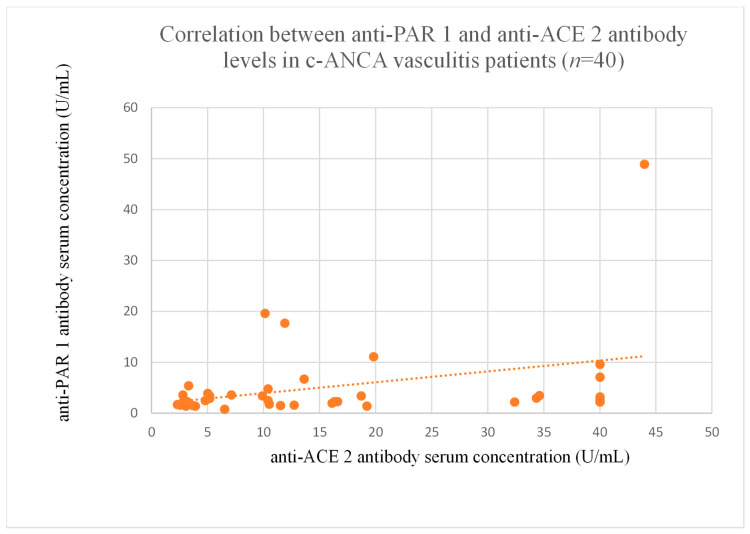
Correlation between anti-PAR 1 and anti-ACE 2 antibody levels in c-ANCA vasculitis patients (r = 0.35, *p* = 0.02).

**Figure 23:**
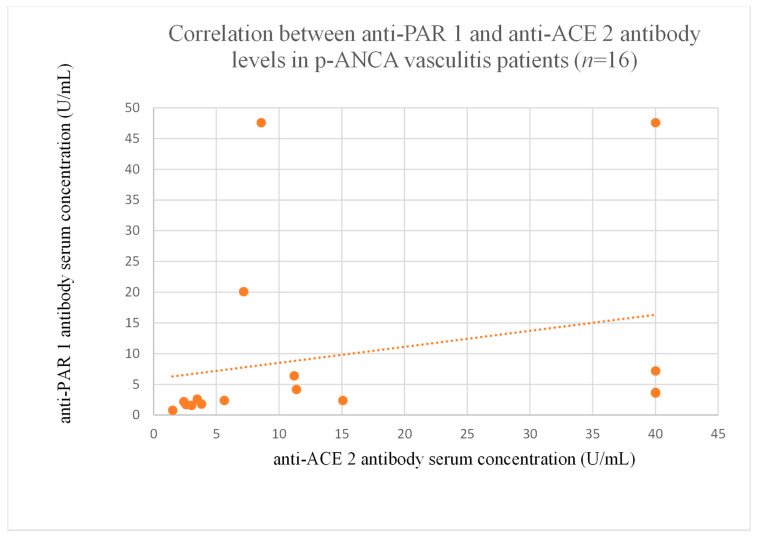
Correlation between anti-PAR 1 and anti-ACE 2 antibody levels in p-ANCA vasculitis patients (r = 0.71, *p* = 0.002).

The correlations between anti-PAR 1 and anti-ACE 2 antibody levels were significant in the membranous nephropathy (Figure 20), focal and segmental glomerulosclerosis (Figure 21), c-ANCA-positive vasculitis (Figure 22), and p-ANCA-positive vasculitis groups (Figure 23).

## 4. Discussion

Anti-PAR 1 and anti-ACE 2 antibodies correlated positively with protein and albumin levels in FSGS at different time points, with higher levels being associated with a less severe disease course (Figure 4, Figure 5 and Figure 6, Figure 12, Figure 13, Figure 14, Figure 15 and Figure 16). In many groups, only one of two factors—total protein or albumin—showed a correlation with the antibody levels; in fact, the second factor’s correlation with antibodies was also nearly statistically significant. Furthermore, those with FSGS had lower anti-PAR 1 antibodies than healthy controls (Figure 2). These observations are consistent and suggest the existence of a mechanism in FSGS that lowers antibody levels. Interestingly, our study on the anti-ETAR antibody also found diminished antibody levels in FSGS [1]. Although the mechanism behind this phenomenon is unknown, it is probably related to FSGS pathophysiology, in which some parts of the glomeruli are destroyed, and others are sclerosed [33]. Inflammation may be suppressed in the sclerosed parts of the glomeruli, leading to reduced autoantibody production. Alternatively, lowered antibody production may result from the binding of high levels of antigens generated during the course of the disease. Further work should be conducted using molecular models to test these hypotheses.

Anti-PAR 1 antibody levels were lower in the membranous nephropathy group compared to the healthy controls, perhaps due to the binding of antibodies to antigens throughout the disease course [34]. However, there were no significant correlations between anti-PAR 1 and the clinical course of the disease, which may be due to the low number of observations made or the nature of antigens. Indeed, some antigens may bind more often with their antibodies, and this binding could be resistant to fluctuations over time [35]. Treatment could also have impacted these findings, with variability in the response of the antigens and antibodies perhaps explaining the differences between types of glomerulonephritis.

Anti-PAR 1 (Figure 7, Figure 8, Figure 9 and Figure 10) and anti-ACE 2 (Figure 17 and Figure 18) correlated negatively with total protein and albumin levels in LN and were higher in LN than in membranous nephropathy, FSGS, IgA nephropathy, non-IgA mesangial proliferative glomerulonephritis, and c-ANCA-positive vasculitis (Figure 2 and Figure 3). Furthermore, higher levels of autoantibodies were linked to a more severe LN course. Systemic lupus erythematosus is a very active autoimmune disease that produces many autoantibodies, which is consistent with our findings of elevated autoantibodies in LN [36]. Such antibodies likely represent a spectrum of those found in systemic lupus erythematosus.

While many correlations between antibody levels and total protein were statistically significant in this study, correlations between antibody levels and albumin were in some cases not statistically significant, but they were almost so. Even though albumin is a better indication of illness severity in these cases, total protein still shows some general trends. The amount of albumin and total protein are related. Similar to correlations between antibody levels and total protein, correlations between antibody concentration and albumin are likely to be statistically significant in larger patient groups.

Correlations were found between anti-PAR 1 and anti-ACE 2 antibodies and creatinine in IgA nephropathy patients (Figure 19). However, since this finding only occurred at a single time point, the antibodies may have limited influence due to treatments and the relatively low number of observations. Moreover, correlations between antibodies and eGFR, and BUN did not reach statistical significance.

The correlations between anti-PAR 1 and anti-ACE 2 antibodies were significant in membranous nephropathy (Figure 20) and FSGS (Figure 21), with lower anti-PAR 1 levels than the control group (Figure 2). Moreover, correlations were marked between both antibodies and the clinical course of FSGS. These findings suggest a role for these antibodies in FSGS pathogenesis and indicate that anti-PAR 1 and anti-ACE 2 could be useful prognostic markers and that the pathways of the two are connected throughout FSGS development. Moreover, the observations are consistent with research showing that PAR 1 activation induced an FSGS model in mice [18]. Some researchers suggest that PAR 1 may be important for maintaining a proper glomerular filtration barrier, but they were unsure [19]. Our study seems to support this thesis.

Although positive correlations were found between both antibodies in c-ANCA-positive (Figure 22) and p-ANCA-positive vasculitis (Figure 23), there was no significant influence on disease course, and the levels were similar to that of healthy controls.

The results of the anti-PAR 1 antibody evaluation were similar between the healthy control group (median = 2.8 (range: 1.5–47.6) +/−10.8 U/mL) and the findings of a previously published study (median = 2.4 (range: 1.99–4.7) U/mL) [23].

Higher anti-PAR 1 antibody level in secondary glomerulonephritis in comparison to primary glomerulonephritis probably reflects the systemic effect of systemic diseases with the production of many autoantibodies circulating in the blood. Anti-ACE 2 antibodies did not differ between the groups, so it means that the systemic production of these two kinds of antibodies do not go together in the evaluated systemic diseases or that the evaluated groups were too small.

Evaluation of anti-PAR 1 antibodies in systemic diseases and their connections with other pathways may be a promising direction, as, lastly, one group of researchers found association between anti-PAR 1 antibodies and endothelin 1 in patients with scleroderma renal crisis [37].

### 4.1. Study Limitations

Material collection was performed before starting treatment with ACE inhibitors, the standard treatment for patients with proteinuria and glomerulonephritis. ACE inhibitors can influence the active levels of the enzyme, which may change anti-ACE 2 antibody concentrations. The correlations between baseline anti-ACE 2 clinical values primarily occurred over a short period (one, three, and six months), and it is probable that long-term observations would demonstrate a more substantial influence of ACE inhibitor treatment.

During the observation period, membranous glomerulonephritis, FSGS, and non-IgA mesangial proliferative glomerulonephritis patients were administered standard immunosuppression consisting of three doses of methylprednisolone (500 mg) followed by oral prednisone (1 mg/kg) with subsequent dose reduction. Patients with IgA nephropathy were treated with three doses of methylprednisolone (500 mg) every two months (three times). Among pulses of methylprednisolone, they also received prednisone (0.5 mg/kg) every other day for six months. LN and vasculitis treatment included daily azathioprine (100 mg), cyclophosphamide (500 mg) every two weeks (six times), and an initial dose of prednisone (1 mg/kg) that was reduced over time.

The range of anti-PAR 1 in our control group was very wide (1.5–47.6 U/mL), which weakens the significance of our findings. Nevertheless, despite that, statistical differences were still important. The control group, despite a normal creatinine range, had a relatively low eGFR. From the other side, eGFR in the control group did not differ from the eGFR in focal and segmental glomerulosclerosis and membranous nephropathy, so one cannot say that low antibodies in these nephropathies are connected with low eGFR. It seems, rather, that this diminishment of antibody levels is connected with the diagnosis.

The findings of the current study require confirmation in a larger population of subjects and further evaluation using a prospective cohort design. Furthermore, anti-PAR 1 and anti-ACE 2 antibody activities in specific glomerular diseases need further exploration using molecular models. Additional work should involve a meta-analysis of studies and models using artificial intelligence to gain a comprehensive understanding of the associations between different markers, pathways, and antibodies in glomerular diseases.

### 4.2. Future Perspectives

Anti-PAR 1 and anti-ACE 2 antibodies could be useful prognostic markers for FSGS and LN over a 0–6-month timeframe. Assessing the levels of these antibodies is straightforward and only requires serum. Such tests could lessen the necessity for kidney biopsy and may be especially useful when combined with similar tests for other antibodies, such as anti-ETAR and anti-C-X-C motif chemokine receptor 3 (CXCR3) [1].

Future studies should focus on the interactions between the antibodies and drugs, such as ACE inhibitors, the ACE 2 activator xanthenon [38], and the PAR 1 inhibitor voraxapar [39]. Indeed, the therapeutic inhibition of anti-PAR 1 can cause PAR 1 fluctuations that have been shown to deteriorate kidney function [19]. Since PAR 1 inhibitors can alter PAR 1 and anti-PAR 1, clinical outcomes may depend on interactions between the three.

Nonetheless, our study is the first to evaluate anti-PAR 1 and anti-ACE 2 antibody levels in glomerular disease and we found an association between antibody levels and the clinical course of FSGS and LN.

## 5. Conclusions

Anti-PAR 1 antibodies were lower in focal and segmental glomerulosclerosis and membranous nephropathy patients compared to the healthy control group.

Low anti-PAR 1 antibody levels seem to be connected with lower levels of total protein and albumin in focal and segmental glomerulosclerosis patients.

Higher levels of anti-PAR 1 antibodies tend to be connected with lower levels of total protein and albumin in the lupus nephritis group.

Anti-ACE 2 antibodies had the same tendencies, but less pronounced.

Levels of anti-PAR 1 antibodies seem to be higher in secondary compared to primary glomerulonephritis. Our results require confirmation using bigger groups of patients.

## Figures and Tables

**Table 1:** Clinical data of patients with specific glomerular diseases (median values).

Diagnosis	Serum Creatinine (mg/dL)	eGFR (mL/min./1.73 m^2^) MDRD	BUN (mg/dL)	Proteinuria (mg/per day)	Albumin to Creatinine Ratio	Total Serum Protein (g/dL)	Serum Albumin (g/dL)
membranous nephropathy (*n* = 18)	1.25 (0.8–3.3)	66 (15–106)	12 (8–32)	2640 (100–1580)	1.6 (0.3–7.1)	4.8 (3.7–5.9)	2.8 (1.7–3.9)
focal and segmental glomerulosclerosis (*n* = 25)	1.21 (0.73–3.19)	68 (26–126)	12 (8–30)	2300 (70–13,990)	1.3 (0.3–7.5)	5 (3–7.3)	2.9 (1.3–4.8)
lupus nephritis (*n* = 17)	1.06 (0.77–2.19)	68 (31–116)	9 (7–23)	1590 (180–5950)	0.8 (0.3–3.1)	5.5 (3.8–7.3)	3.1 (2–4)
IgA nephropathy (*n* = 14)	1.06 (0.71–1.82)	70 (35–131)	9.5 (7–20)	940 (90–4540)	0.6 (0.3–2.2)	5.65 (4.4–6.5)	3.4 (2.2–4)
mesangial proliferative (non-IgA) glomerulonephritis (*n* = 6)	0.93 (0.59–1.55)	105 (40–131)	8.5 (6–16)	2580 (620–7130)	0.8 (0.4–2.9)	4.8 (3.9–5.2)	2.8 (1.6–3.2)
control group (*n* = 22)	1.2 (0.9–1.3)	63 (60–78)	12 (9–16)	0 (0–0)	0 (0–0)	7.4 (6.6–8.2)	4.4 (3.5–5.2)
c-ANCA vasculitis (*n* = 40)	1.81 (0.69–7.78)	28 (7–126)	19.8 (7–75)	640 (60–19,000)	0.4 (0.3–10.9)	6.3 (5.3–7.1)	3.6 (2.4–4.6)
p-ANCA vasculitis (*n* = 16)	3.13 (0.79–9.04)	19 (5–93))	30 (8–81)	1730 (140–12,300)	0.8 (0.3–5)	5.95 (4.8–8.3)	3.5 (2.8–4.3)

**Table 2:** Clinical data of patients with specific glomerular diseases (median values).

Diagnosis	Age (Years)	Sex (Percent of Females)	Hb (g/dL)	HCT (%)	Leukocytes (Number/Microlitre)
membranous nephropathy (*n* = 18)	51.5 (28–69)	45%	13.5 (11.3–16)	44.5 (38–52.6)	6715 (2500–10,780)
focal and segmental glomerulosclerosis (*n* = 25)	48 (19–74)	44%	13.9 (9.3–18)	45.7 (36.8–55.2)	7690 (4130–10,500)
lupus nephritis (*n* = 17)	34 (19–66)	53%	13.2 (10.5–17.3)	43 (34.5–56.9)	6600 (4090–10,900)
IgA nephropathy (*n* = 14)	45.5 (20–60)	50%	14.8 (12.2–16.5)	48.6 (46–54.3)	8420 (5360–10,900)
mesangial proliferative (non-IgA) glomerulonephritis (*n* = 6)	28 (20–52)	50%	14.8 (10.1–18)	45.2 (33.2–55)	10,200 (6200–10,490)
control group (*n* = 22)	44 (26–80)	50%	14.6 (12.3–16.9)	45.7 (37.9–53)	6270 (4280–7780)
c-ANCA vasculitis (*n* = 40)	58 (21–81)	55%	12 (7.7–16.1)	37.3 (27–49.7)	6555 (3740–10,450)
p-ANCA vasculitis (*n* = 16)	62 (37–87)	44%	9.6 (8.3–13.2)	30.4 (28.4–42)	4905 (3240–9620)

## Data Availability

Data are contained within the article or Supplementary Materials. The original contributions presented in this study are included in the article/Supplementary Materials. Further inquiries can be directed to the corresponding author(s).

## Supplementary Materials

The following are available online at https://www.mdpi.com/article/10.3390/jcm14093178/s1, Figure S1: Values of serum anti-PAR 1 antibody level in specific groups of patients (U/mL), Figure S2: Values of serum anti-ACE 2 antibody level in specific groups of patients (U/mL).

## Abbreviations

List of Abbreviations (In Alphabetical Order)

ACE	angiotensin 2-converting enzyme type 1
ACE 2	angiotensin 2-converting enzyme type 2
ANAs	antinuclear antibodies
ANOVA	analysis of variance
AT1R	angiotensin 2 type 1 receptor
AT2R	angiotensin 2 type 2 receptor
cANCA	anti-neutrophil cytoplasmic antibodies
ELISA	enzyme-linked immunosorbent assay
ETAR	endothelin A receptor
FSGS	focal and segmental glomerulosclerosis
HLA	human leukocyte antigens
LN	lupus nephritis
PAR 1	protease-activated receptor type 1
pANCA	perinuclear anti-neutrophil cytoplasmic antibodies
POD	horseradish peroxidase
TMB	tetramethylbenzidine
