# Supplementary figures and images for: The Influence of Anti-PAR 1 and Anti-ACE 2 Antibody Levels on the Course of Specific Glomerulonephritis Types
Source: J Clin Med. 2025 May 4;14(9):3178. doi: 10.3390/jcm14093178 (PMC12072768; doi:10.3390/jcm14093178)

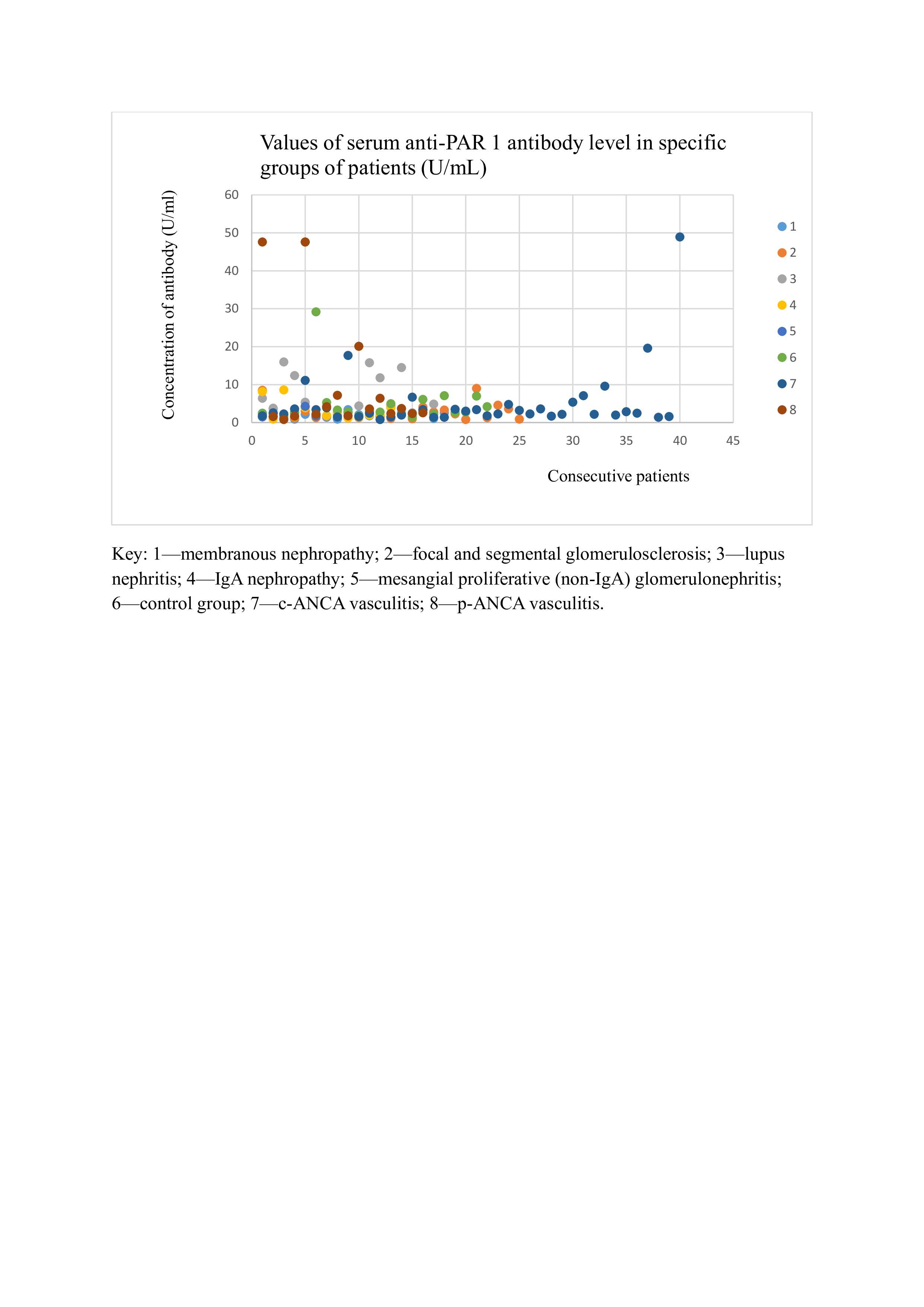

Supplement: Supplementary file 1 [file jcm-14-03178-s001.zip › Figure S1.jpg]

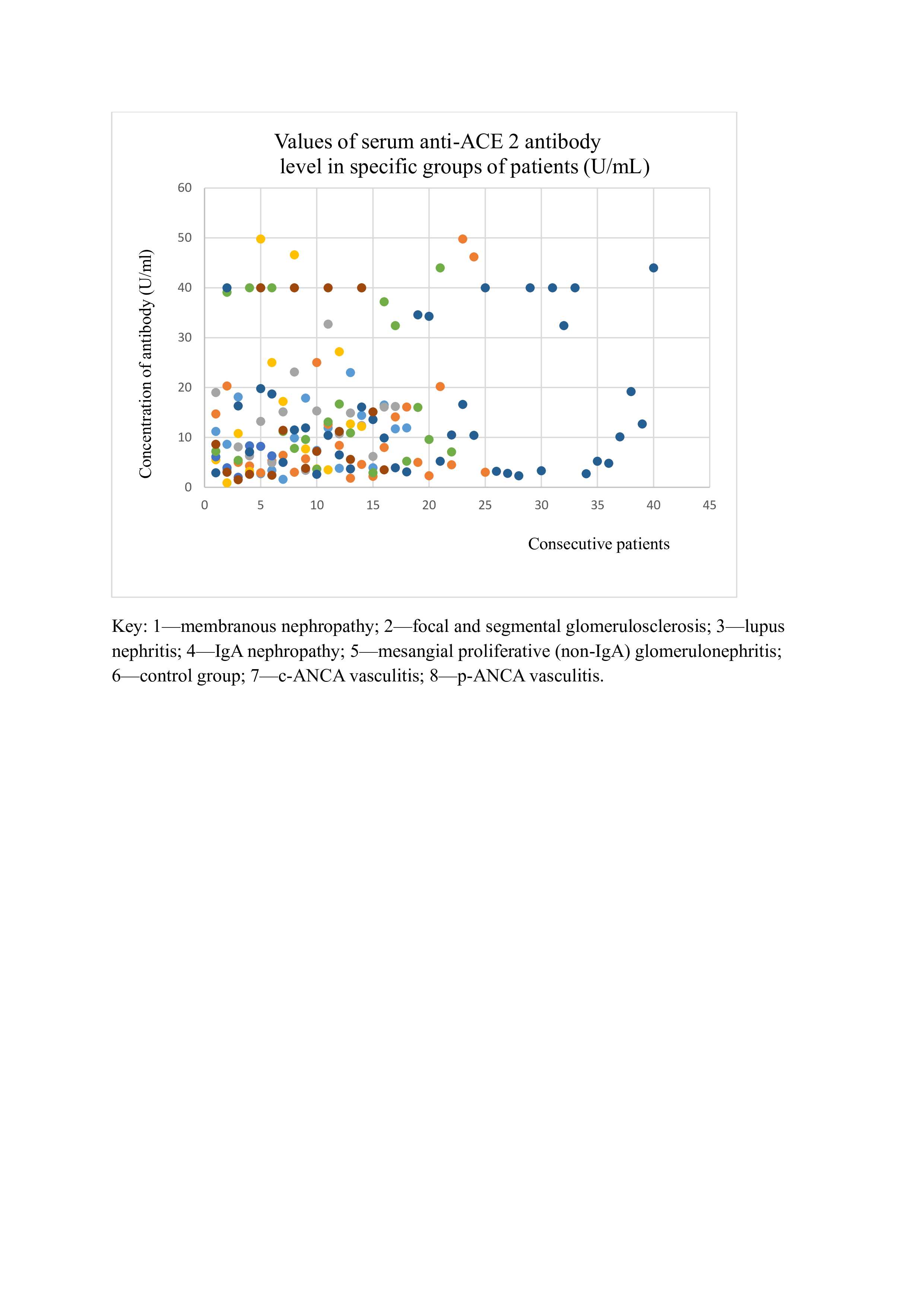

Supplement: Supplementary file 1 [file jcm-14-03178-s001.zip › Figure S2.jpg]
